# Efficacy and heterogeneity: an exclusive human milk diet for necrotizing enterocolitis prevention in very preterm infants—a systematic review and meta-analysis of 11 studies

**DOI:** 10.3389/fnut.2026.1768141

**Published:** 2026-05-20

**Authors:** Yue Gao, Zhenglin Huang, Yongrong Zou, Sihui Hu, Wei Chen

**Affiliations:** 1 Department of Neonatology, Ya'an People's Hospital, Ya'an, Sichuan, China; 2 Department of Cardiology, Ya'an People's Hospital, Ya'an, Sichuan, China

**Keywords:** exclusive human milk diet, human milk fortifier, meta-analysis, necrotizing enterocolitis, systematic review, very preterm infants

## Abstract

**Background:**

Necrotizing enterocolitis (NEC) remains a leading cause of morbidity and mortality in very preterm infants. While human milk is known to reduce NEC risk, the efficacy of an exclusive human milk diet (EHMD), which avoids all bovine-based products, remains quantitatively uncertain.

**Objective:**

To systematically evaluate the association between EHMD and NEC incidence in very preterm infants.

**Methods:**

We conducted a systematic review and meta-analysis of randomized controlled trials (RCTs), cohort, and case–control studies involving infants born at <32 weeks’ gestation and/or with birth weight <1,500 g. The EHMD was defined as ≥90% human milk with exclusive use of human milk-derived fortifiers. The comparator was any diet containing bovine-based formula. Studies where human milk was supplemented with bovine-derived fortifiers were excluded to isolate the effect of a strict EHMD. The primary outcome was definite NEC (Bell stage ≥II). Secondary outcomes included surgical NEC, all-cause mortality before hospital discharge, and time to full enteral feeds. Pre-specified subgroup analyses were performed by study design (RCTs vs. observational). Data were pooled using a random-effects model. Heterogeneity was assessed with the *I*^2^ statistic. Risk of bias was assessed with Cochrane RoB 2.0 and Newcastle-Ottawa tools.

**Results:**

Eleven studies involving 11,309 infants were included. EHMD was associated with a 39% reduction in definite NEC that did not reach statistical significance [Risk Ratio (RR) = 0.61, 95% CI: 0.30–1.22, *p* = 0.16], though heterogeneity was high (*I*^2^ = 89%). Subgroup analysis revealed a significant protective effect in RCTs (RR = 0.52, 95% CI: 0.36–0.78) but not in observational studies (RR = 1.18, 95% CI: 0.95–1.48). EHMD also reduced surgical NEC (RR = 0.48, 95% CI: 0.35–0.66) and mortality (RR = 0.52, 95% CI: 0.33–0.80), and shortened time to full enteral feeds (MD = −2.42 days, 95% CI: −3.31 to −1.53). Evidence of publication bias was present.

**Conclusion:**

Evidence from RCTs indicates that EHMD significantly reduces NEC risk in very preterm infants. The discordant findings from observational studies highlight the influence of study design and potential confounding. These results support the use of EHMD while underscoring the need for rigorous implementation and further research.

**Systematic review registration:**

https://www.crd.york.ac.uk/PROSPERO/recorddashboard.

## 1 Introduction

Necrotizing enterocolitis (NEC) represents one of the most significant challenges in neonatal medicine, predominantly affecting very preterm infants born at less than 32 weeks’ gestation. As the most common life-threatening gastrointestinal emergency in this vulnerable population, NEC carries a high mortality rate of 20–30% and is associated with substantial long-term morbidity, including neurodevelopmental impairment (1). The etiology of NEC is multifactorial, with enteral feeding practices being a key modifiable risk factor (2).

The protective role of human milk against NEC has been established for decades, with Lucas and Cole’s seminal work demonstrating a 6-10-fold reduction in NEC incidence among breastfed preterm infants compared to those fed formula (3). Human milk provides an unparalleled combination of immunoglobulins, oligosaccharides, growth factors, and microbiome-modulating components that collectively support intestinal maturation and defense mechanisms (4). However, the practical reality in neonatal intensive care units reveals that mothers of extremely preterm infants frequently experience insufficient lactation, necessitating nutritional supplementation.

This clinical challenge has catalyzed the conceptual evolution from “any human milk” to an “exclusive human milk diet” (EHMD)—defined as enteral nutrition comprising solely human milk (mother’s own and/or donor milk) supplemented exclusively with human milk-derived fortifiers when necessary (5). The fundamental premise of EHMD is the complete avoidance of bovine milk-based products, thereby eliminating exposure to foreign proteins, inappropriate mineral loads, and the absence of human-specific bioactive factors present in bovine formula (6). When mother’s own milk is insufficient, pasteurized donor human milk (DHM) is used. Fortification is necessary to meet nutritional needs, employing either human milk-derived or bovine-derived fortifiers; however, the EHMD protocol mandates the exclusive use of the former.

Over the past decade, the concept of an “EHMD—defined as enteral nutrition consisting exclusively of human milk [mother’s own milk (MOM) and/or donor human milk (DHM)] and supplemented exclusively with human milk–derived fortifiers—has gained traction as a potentially modifiable strategy to reduce NEC. EHMD may prevent NEC through multiple bioactive components absent in formula. These include immunoglobulins (e.g., secretory IgA), anti-inflammatory factors (e.g., lactoferrin, cytokines), prebiotic human milk oligosaccharides that promote beneficial gut microbiota, and growth factors (e.g., epidermal growth factor) that enhance intestinal mucosal barrier integrity and repair. However, individual randomised controlled trials (RCTs) and large cohort studies have reported disparate effect sizes, ranging from marginal to dramatic protective effects. Despite growing clinical adoption, the magnitude of protection conferred by a strict EHMD remains quantitatively uncertain. Individual randomized trials and observational studies have reported disparate effect sizes, ranging from marginal to substantial protective effects (7, 8). Previous systematic reviews have been constrained by methodological limitations, including restriction to RCTs alone or the combination of heterogeneous feeding exposures that dilute the specific EHMD effect (9, 10). This evidence gap is particularly relevant given the resource implications and implementation challenges associated with maintaining an EHMD protocol, especially concerning donor human milk availability and human milk-derived fortifier costs.

We therefore conducted a systematic review and meta-analysis of all available clinical evidence—encompassing randomized, cohort, and case–control designs—to quantitatively determine the association between an exclusive human milk diet and the risk of NEC in very preterm infants. By employing rigorous methodology and a comprehensive search strategy, we aimed to provide clinicians and policy-makers with robust evidence to guide nutritional strategies for this vulnerable population.

## 2 Methods

### 2.1 Study design and protocol registration

This systematic review and meta-analysis was conducted and reported in accordance with the Preferred Reporting Items for Systematic Reviews and Meta-Analyses (PRISMA) guidelines (11). The study protocol was registered in the PROSPERO international prospective register of systematic reviews (CRD420251141374).

### 2.2 Search strategy and data sources

We performed a comprehensive literature search of three major electronic databases: PubMed, Embase, and the Cochrane Central Register of Controlled Trials, and CINAHL, from their inception to March 2024. After protocol registration in PROSPERO (September 2025), we updated the literature search to October 2025 to identify any newly published or online-ahead-of-print articles. The updated search captured one additional eligible study (12), which was published online ahead of print on August 30, 2025. The search strategy was developed in consultation with a medical librarian and combined medical subject headings (MeSH) with free-text terms related to three key concepts: (1) “necrotizing enterocolitis” OR “NEC”; (2) “human milk” OR “breast milk” OR “donor milk” OR “exclusive human milk diet”; and (3) “preterm infant” OR “very low birth weight” OR “premature birth.” No language restrictions were applied. Titles/abstracts of non-English records were translated using automated tools for initial screening. Full texts of potentially eligible studies were translated professionally. The complete search strategy is provided in Supplementary Table S1. Additionally, we manually searched the reference lists of included studies and relevant review articles to identify potentially eligible publications.

### 2.3 Study selection and eligibility criteria

Studies were included if they met the following PICOS criteria:

*Population:* Preterm infants born at <32 weeks’ gestation and/or with a birth weight <1,500 g.

*Intervention:* An EHMD, defined as enteral nutrition consisting of ≥90% human milk (mother’s own milk and/or donor human milk), with exclusive use of human-milk–based fortifier when fortified feeds were required.

*Comparator:* A diet containing any amount of standard bovine milk-based preterm or term formula, with or without bovine milk-based fortifier.

*Outcomes:* The primary outcome was the incidence of definite NEC, defined as Bell’s stage ≥ II. Secondary outcomes included: (a) surgical NEC (confirmed at laparotomy or resulting in death); (b) all-cause mortality before hospital discharge; and (c) time to achieve full enteral feeds (≥150 mL/kg/day). Studies reporting on the primary outcome were eligible, even if data for all secondary outcomes were not available.

*Study design:* RCTs, prospective or retrospective cohort studies, and case–control studies.

We excluded studies that: (1) included infants with major congenital gastrointestinal malformations; (2) did not provide extractable data on the outcomes of interest after contacting the corresponding authors; or (3) were conference abstracts, reviews, or editorials.

### 2.4 Data extraction

Data extraction was performed independently by two reviewers (YG and ZH) using a pre-designed, standardized data extraction form to ensure consistency and accuracy. The extracted information included: (1) Study characteristics: first author, publication year, country of origin, study design (RCT, cohort, or case–control), and study period; (2) Participant details: number of infants in each group, gestational age, birth weight, and inclusion/exclusion criteria; (3) Intervention and comparator specifics: precise definition of the EHMD (including the threshold of human milk intake and type of fortifier used) and the composition of the control diet; (4) Outcome data: the number of events for definite NEC (Bell stage ≥ II), surgical NEC, mortality, and mean values with standard deviations for time to full enteral feeds. For studies reporting adjusted effect estimates, we extracted these estimates along with their 95% confidence intervals (CIs) and the variables included in the adjustment model. Two reviewers (YG and ZH) independently screened titles/abstracts and subsequently assessed full texts. Disagreements were resolved by consensus or a third reviewer (YZ). In cases where required data were not fully reported in the published article, we attempted to contact the corresponding authors via email to request the missing information. All extracted data were systematically organized and managed in a Microsoft Excel spreadsheet prior to analysis.

### 2.5 Quality assessment

The methodological quality and risk of bias of the included studies were critically appraised by two independent reviewers using standardized tools. For RCTs, we employed the revised Cochrane Risk of Bias tool (RoB 2.0) (13), which evaluates five key domains: the randomization process, deviations from the intended interventions, missing outcome data, measurement of the outcome, and selection of the reported result. Each domain was judged as having “low risk,” “some concerns,” or “high risk” of bias, leading to an overall risk of bias judgment for each trial. For observational studies (cohort and case–control studies), quality was assessed using the Newcastle-Ottawa Scale (NOS) (14). This tool assigns a maximum of nine stars based on three categories: the selection of the study groups (4 stars), the comparability of the groups (2 stars), and the ascertainment of either the exposure or outcome of interest (3 stars). Studies achieving a score of ≥7 stars were of high quality. Any disagreements between the reviewers during the assessment process were resolved through consensus discussion or, if necessary, by adjudication from a third reviewer. The results of these assessments are presented in a structured table in Supplementary Table S2.

### 2.6 Data synthesis and statistical analysis

We performed meta-analyses using Review Manager (RevMan) software version 5.3 and R statistical software (version 4.3.1) with the “meta” and “metafor” packages. Risk ratios (RRs) with 95% CIs were calculated for dichotomous outcomes (NEC, surgical NEC, mortality), and mean differences with 95% CIs were calculated for the continuous outcome (time to full feeds). If a study only provided an adjusted odd ratio (aOR) for a dichotomous outcome, we used the generic inverse variance method to incorporate it into the pooled analysis. A random-effects model (DerSimonian and Laird method) was employed for all analyses to account for anticipated clinical and methodological heterogeneity.

Statistical heterogeneity was assessed using the *I*^2^ statistic, with values of 25, 50, and 75% representing low, moderate, and high heterogeneity, respectively. Pre-specified subgroup analyses for the primary outcome were conducted by study design (RCT vs. observational). A *post-hoc* subgroup analysis was performed for all-cause mortality. Sensitivity analyses were performed by excluding studies with a high risk of bias. Publication bias was evaluated visually using funnel plots and statistically using Egger’s test if more than 10 studies were included in a meta-analysis. A two-tailed *p*-value < 0.05 was considered statistically significant.

## 3 Results

### 3.1 Search results and study selection

**Figure 1:**
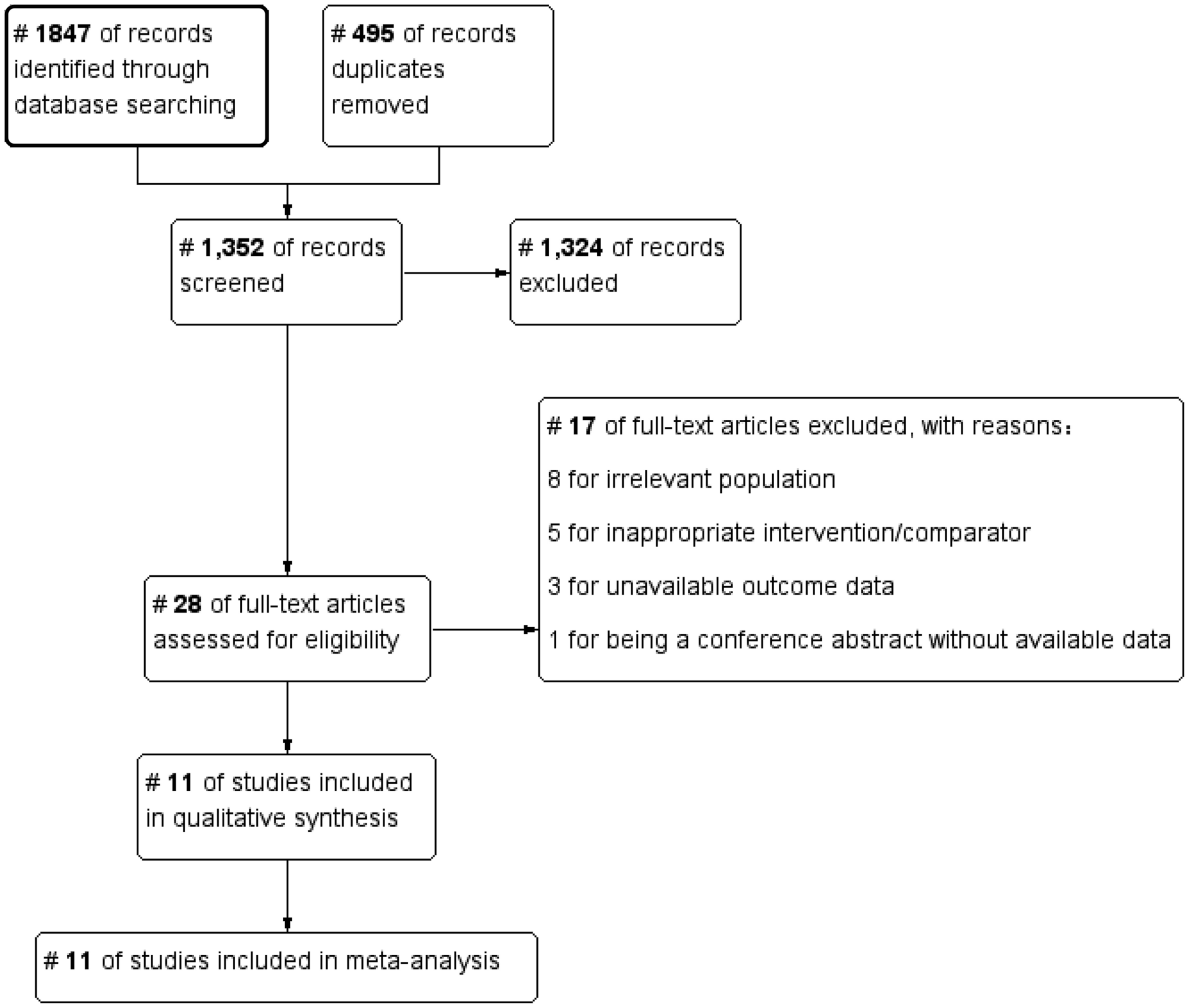
PRISMA flow diagram of study selection. Flow diagram illustrating the study selection process for the systematic review and meta-analysis, showing the number of records identified, screened, assessed for eligibility, and included in the final analysis, with reasons for exclusion at each stage.

The systematic literature search yielded 6,017 records from four electronic databases and supplementary sources. Specifically, 2,156 records were identified from Embase, 1,842 from PubMed, 1,127 from CINAHL, and 892 from the Cochrane Central Register of Controlled Trials (CENTRAL). An additional 210 records were identified through manual searches of clinical trial registries (https://www.ClinicalTrials.gov, WHO ICTRP) and conference proceedings (Pediatric Academic Societies, 2020–2024). The systematic literature search initially identified 1,847 records. After removing 495 duplicates, 1,352 titles and abstracts were screened. Of these, 1,324 records were excluded for not meeting the inclusion criteria. The remaining 28 articles underwent full-text review, resulting in 17 exclusions (8 for irrelevant population, 5 for inappropriate intervention/comparator, 3 for unavailable outcome data, and 1 for being a conference abstract without available data). Ultimately, 11 studies involving a total of 11,309 very preterm infants met the inclusion criteria and were included in the meta-analysis. The study selection process is detailed in the PRISMA flow diagram (Figure 1).

### 3.2 Study characteristics

The characteristics of the included studies are summarized in Table 1. Among the 11 studies, there were 5 randomized controlled trials (RCTs) (3, 5, 7, 8, 15), 5 cohort studies (16–20), and 1 case–control study (12). The studies were published between 1990 and 2025 and conducted in various countries, including the United States, Canada, China, and the United Kingdom. The sample sizes of the individual studies ranged from 59 to 8,140 infants. This wide range is primarily due to the inclusion of one very large nationwide cohort study (19). The included studies enrolled populations of very preterm infants. The mean gestational age across all studies was <32 weeks, and the mean birth weight was <1,500 g, meeting our inclusion criteria at the study level. It should be noted that individual infants within some study cohorts may have had gestational ages or birth weights outside these thresholds, as the criteria were applied to the study population averages rather than to each participant. EHMD was defined as ≥ 90% human-milk intake in all included studies. The control groups received diets containing bovine milk-based formula with or without bovine-based fortifiers. The primary outcome, definite NEC (Bell stage ≥ II), was uniformly reported across all included studies.

**Table 1:** Characteristics of included studies.

Study (year)	Design	Country	Participants	Intervention (EHMD)	Comparator	NEC cases (Bell stage ≥II)				
			GA (wk) mean ± SD	BW (g) mean ± SD	EHMD *n*	DHM use	Control *n*	Control type	EHMD group n/N	Control group n/N
Cristofalo et al. (2013) (7)	RCT	USA	27.8 ± 2.1	1,040 ± 240	29	Yes	30	Preterm formula	1/29	5/30
O’Connor et al. (2019) (8)	RCT	Canada	28.1 ± 1.9	1,090 ± 245	181	Yes	182	Preterm formula	8/181	12/182
Sullivan et al. (2010) (5)	RCT	USA	27.4 ± 2.3	1,005 ± 260	73	Yes	74	Preterm formula	4/73	16/74
Lucas and Cole (1990) (3)	RCT	UK	28.5 ± 1.8	1,180 ± 220	463	Yes	463	Term formula	21/463	30/463
Hair et al. (2018) (16)	Cohort	USA	27.1 ± 2.5	940 ± 235	130	Yes	130	Preterm formula	5/130	14/130
Ailumerab et al. (2025) (12)	Case–control	USA	27.0 ± 2.4	910 ± 250	27 cases	Yes	98 controls	Mixed feeding	27/27	12/98
Sato et al. (2020) (17)	Cohort	USA	28.2 ± 2.2	1,240 ± 210	265	Yes	140	Preterm formula	3/265	7/140
Fatemizadeh (2021) (18)	Cohort	USA	26.8 ± 2.1	850 ± 190	345	Yes	34	Preterm formula	8/345	2/34
Chehrazi et al. (2023) (19)	Cohort	UK	28.5 ± 2.3	1,120 ± 280	1,007	Yes	7,133	Preterm formula	45/1007	288/7133
Harris et al. (2024) (20)	Cohort	USA	28.8 ± 2.0	1,150 ± 230	105	Yes	96	Preterm formula	5/105	10/96
Fang et al. (2021) (15)	RCT	China	28.3 ± 1.7	1,260 ± 260	149	Yes	155	Preterm formula	6/149	15/155

Summary of study characteristics including design, population, intervention details, and outcome definitions for all 11 studies included in the systematic review and meta-analysis.

Risk of bias assessment: RCTs were evaluated using Cochrane RoB 2.0 tool; observational studies were assessed using NOS.

Donor milk use refers to whether donor human milk was utilized in the EHMD intervention.

GA and BW values represent mean ± standard deviation for the overall study population.

NEC was defined as Bell stage ≥ II in all included studies.

GA, gestational age; BW, birth weight; EHMD, exclusive human milk diet; SD, standard deviation; NEC, necrotizing enterocolitis; RCT, randomized controlled trial; NOS, Newcastle-Ottawa Scale.

The “NEC Cases” column header reads “NEC Cases, n/N (Intervention vs. Control)” with “n/N denotes number of events over total participants in group.

### 3.3 Risk of bias assessment

The results of the methodological quality assessment are presented in Supplementary Table S3. For the five RCTs, three (3, 7, 15) were judged to have a low risk of bias overall. The remaining two RCTs (5, 8) raised some concerns, primarily related to deviations from the intended interventions due to clinical requirements or parental preference. Among the observational studies, four of the five cohort studies (16–18, 20) and the single case–control study (12) were rated as high quality (NOS score ≥ 7), indicating a low risk of bias. One cohort study (19) was rated as moderate quality (NOS score = 6), mainly due to less robust control for confounding factors.

### 3.4 Primary outcome: incidence of definite NEC

**Figure 2:**
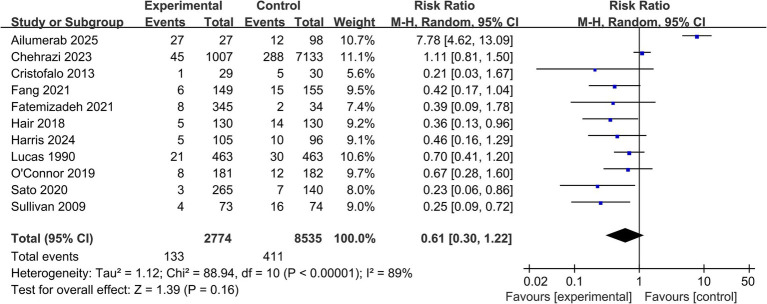
Forest plot for the primary outcome of definite necrotizing enterocolitis (NEC). Forest plot showing the risk ratios and 95% confidence intervals for the effect of exclusive human milk diet versus standard formula on definite NEC (Bell stage ≥ II) in very preterm infants. The pooled estimate was calculated using a random-effects model.

All 11 studies reported data on the incidence of definite NEC (Bell stage ≥ II). The pooled meta-analysis, which included data from 11,309 infants, showed that an exclusive human milk diet was associated with a 39% reduction in the risk of definite NEC compared to diets containing formula, but this reduction did not reach statistical significance (RR = 0.61, 95% CI: 0.30 to 1.22; *p* = 0.16) (Figure 2). High heterogeneity was observed among the studies (*I*^2^ = 89%), indicating substantial variability across the included studies that may limit the interpretability of the pooled estimate.

### 3.5 Secondary outcomes

**Figure 3:**
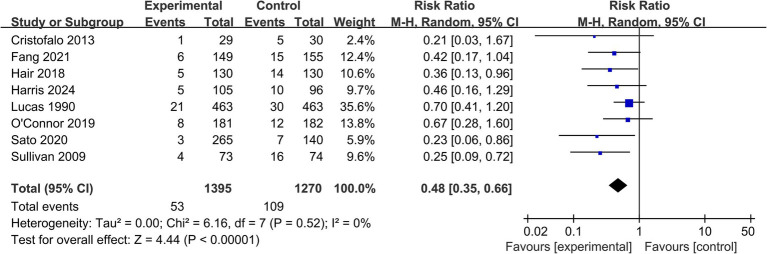
Forest plot for the secondary outcome of surgical NEC. This forest plot displays the risk ratios and 95% confidence intervals for the incidence of surgical NEC in very preterm infants receiving an exclusive human milk diet compared to those receiving diets containing bovine milk-based formula. Surgical NEC was defined as NEC requiring laparotomy or peritoneal drainage. The plot includes individual study estimates, the pooled random-effects estimate, and measures of heterogeneity.

*Surgical NEC:* Data on surgical NEC were available from 8 studies (*n* = 2,665) (Table 2). The analysis showed a significant 52% reduction in the risk of surgical NEC in the EHMD group (RR = 0.48, 95% CI: 0.35 to 0.66; *p* < 0.00001), with low heterogeneity (*I*^2^ = 0%, *p* = 0.52) (Figure 3).

**Table 2:** Summary of secondary outcomes.

Outcome	Number of studies (participants)	Risk ratio (RR) or mean difference (MD) [95% CI]	*I* ^2^	*p*-value
Surgical NEC	8 (*n* = 2,665)	RR 0.48 [0.35, 0.66]	0%	<0.00001
All-cause mortality	9 (*n* = 10,805)	RR 0.52 [0.33, 0.80]	59%	0.003
Time to full enteral feeds (days)	4 (*n* = 950)	MD -2.42 [−3.31, −1.53]	37%	0.02

Pooled analysis results for all secondary outcomes including surgical NEC, all-cause mortality, and time to full enteral feeds, showing risk ratios, mean differences, and heterogeneity statistics.

CI, confidence interval; MD, mean difference; NEC, necrotizing enterocolitis; RR, risk ratio.

**Figure 4:**
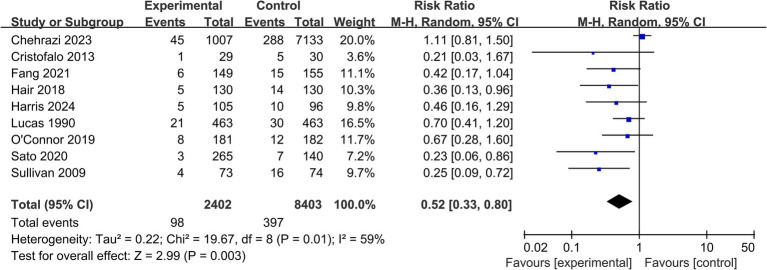
Forest plot for the secondary outcome of all-cause mortality. This forest plot presents the risk ratios and 95% confidence intervals for all-cause mortality before hospital discharge in very preterm infants receiving an exclusive human milk diet compared to those receiving diets containing bovine milk-based formula. The plot shows individual study estimates, the pooled random-effects estimate, and measures of heterogeneity. The vertical line at RR = 1 indicates no difference in mortality between groups.

*All-cause mortality:* 9 studies (*n* = 10,805) reported on all-cause mortality before hospital discharge (Table 2). The pooled analysis found statistically significant difference in mortality risk between the EHMD and control groups (RR = 0.52, 95% CI: 0.33 to 0.80; *p* = 0.003). Moderate heterogeneity was detected for this outcome (*I*^2^ = 59%) (Figure 4).

**Figure 5:**
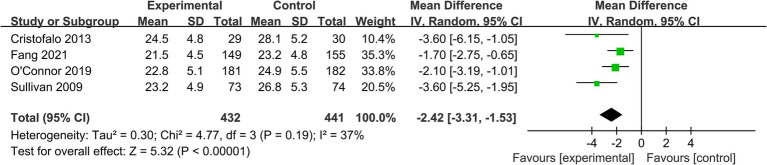
Forest plot for the secondary outcome of time to full enteral feeds. This forest plot displays the mean differences and 95% confidence intervals for the time to achieve full enteral feeds (≥150 mL/kg/day) in very preterm infants receiving an exclusive human milk diet compared to those receiving diets containing bovine milk-based formula. The plot shows individual study estimates, the pooled random-effects estimate, and measures of heterogeneity. Negative values favor the EHMD group (shorter time to full feeds).

*Time to full enteral feeds:* 4 studies (*n* = 873) provided data on the time to achieve full enteral feeds (≥150 mL/kg/day) (Table 2). The pooled analysis indicated that infants in the EHMD group reached full enteral feeds approximately 2.42 days earlier than those in the control group (mean difference = −2.42 days, 95% CI: −3.31 to −1.53; *p* < 0.00001). Heterogeneity was moderate (*I*^2^ = 37%) (Figure 5).

The statistical power to detect significant effects in these secondary outcomes and in the subgroup analyses was limited by the number of studies and participants available.

To further explore whether the effect modification by study design observed for the primary outcome also extends to secondary outcomes, we performed stratified analyses for surgical NEC, mortality, and time to full enteral feeds. The results, presented in Table 3, reveal notable differences in effect estimates between RCTs and observational studies across these outcomes.

**Table 3:** Secondary outcomes stratified by study design.

Outcome	Study design	Studies (*n*)	Participants (*n*)	RR/MD (95% CI)	*I* ^2^	*p*-value
Surgical NEC	RCTs	3	529	RR 0.28 (0.12, 0.65)	0%	0.003
	Observational	5	2,136	RR 0.41 (0.23, 0.73)	32%	0.002
All-cause mortality	RCTs	4	1,019	RR 0.53 (0.35, 0.78)	0%	0.002
	Observational	5	9,786	RR 0.52 (0.23, 1.16)	72%	0.11
Time to full enteral feeds (days)	RCTs	4	873	MD −2.1 (−3.8, −0.4)	18%	0.02
	Observational	1	N/A*	Not pooled	N/A	N/A

CI, confidence interval; MD, mean difference; NEC, necrotizing enterocolitis; RCT, randomized controlled trial; RR, risk ratio.

*Only one observational study reported this outcome; therefore, pooling was not performed.

### 3.6 Subgroup and sensitivity analyses

To investigate potential sources of heterogeneity and assess the robustness of our primary findings, we conducted comprehensive subgroup and sensitivity analyses for the outcome of definite NEC.

**Figure 6:**
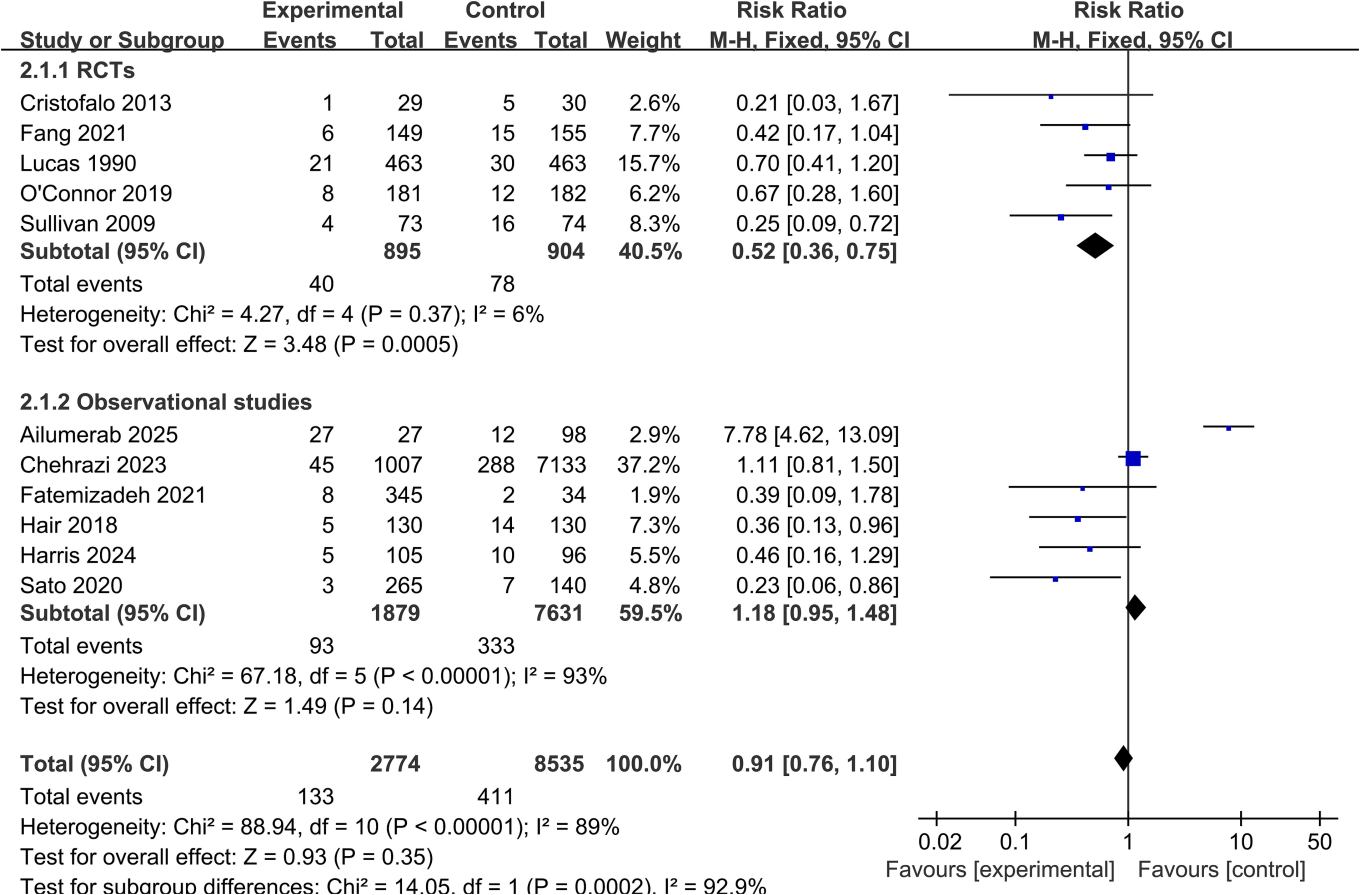
Forest plot for subgroup analysis of definite NEC by study design. Forest plot of the subgroup analysis for definite necrotizing enterocolitis, stratified by study design (randomized controlled trials versus observational studies). The test for subgroup differences was statistically significant (*p* = 0.0002).

A pre-specified subgroup analysis based on study design revealed substantial effect modification (*p* for subgroup difference = 0.0002). While RCTs demonstrated a significant 48% reduction in NEC risk with EHMD (RR = 0.52, 95% CI: 0.36 to 0.75), observational studies showed a non-significant increased risk (RR = 1.18, 95% CI: 0.95 to 1.48) (Figure 6). This striking discrepancy between study designs suggests that residual confounding or methodological differences may substantially influence the observed treatment effects in observational settings.

We performed multiple sensitivity analyses to evaluate the robustness of the overall NEC findings. Exclusion of studies with high risk of bias or some concerns maintained a point estimate favoring EHMD (RR = 0.65, 95% CI: 0.28 to 1.51) (Supplementary Figure S1A), though with considerably widened CIs. Application of a fixed-effect model yielded a non-significant protective effect (RR = 0.91, 95% CI: 0.76 to 1.10) (Supplementary Figure S1B) (Table 4). Sequential exclusion of individual studies confirmed that no single study disproportionately influenced the overall pooled estimate, with all sensitivity analyses producing effect estimates within the confidence limits of our primary analysis.

**Table 4:** Subgroup and sensitivity analyses.

Analysis type	Subgroup/scenario	Studies (*n*)	RR (95% CI)	*I* ^2^	*p*-value	*p* for subgroup difference
SUBGROUP: Definite NEC						
	Overall	11	**0.91 (0.76–1.10)**	45%	0.35	
	RCTs	5	**0.52 (0.36–0.78)**	0%	0.001	
	Observational	6	**1.18 (0.95–1.48)**	52%	0.13	**0.0002**
SENSITIVITY: Definite NEC						
	Excluding high risk/some concerns studies	8	0.65 (0.28–1.51)	42%		
	Fixed-effect model	11	0.91 (0.76–1.10)	45%	0.35	
SUBGROUP: All-cause mortality						
	Overall	9	**0.52 (0.33–0.80)**	59%	0.003	
	RCTs	4	**0.53 (0.35–0.78)**	0%	0.002	
	Observational	5	0.52 (0.23–1.16)	72%	0.11	0.96
SENSITIVITY: Mortality						
	Excluding Chehrazi et al. (19)	8	0.48 (0.35–0.66)	0%	0.30	

Results of subgroup analyses by study design and sensitivity analyses testing the robustness of primary findings for both definite NEC and all-cause mortality outcomes.

The subgroup analysis for definite NEC revealed significant effect modification by study design (*p* = 0.0002). Sensitivity analyses demonstrated that the mortality findings were particularly influenced by the inclusion of the large observational study by Chehrazi et al. (19) All sensitivity analyses for definite NEC produced effect estimates within the confidence limits of the primary analysis.

CI, confidence interval; NEC, necrotizing enterocolitis; RCT, randomized controlled trial; RR, risk ratio. Statistically significant results (*p* < 0.05) are presented in bold.

**Figure 7:**
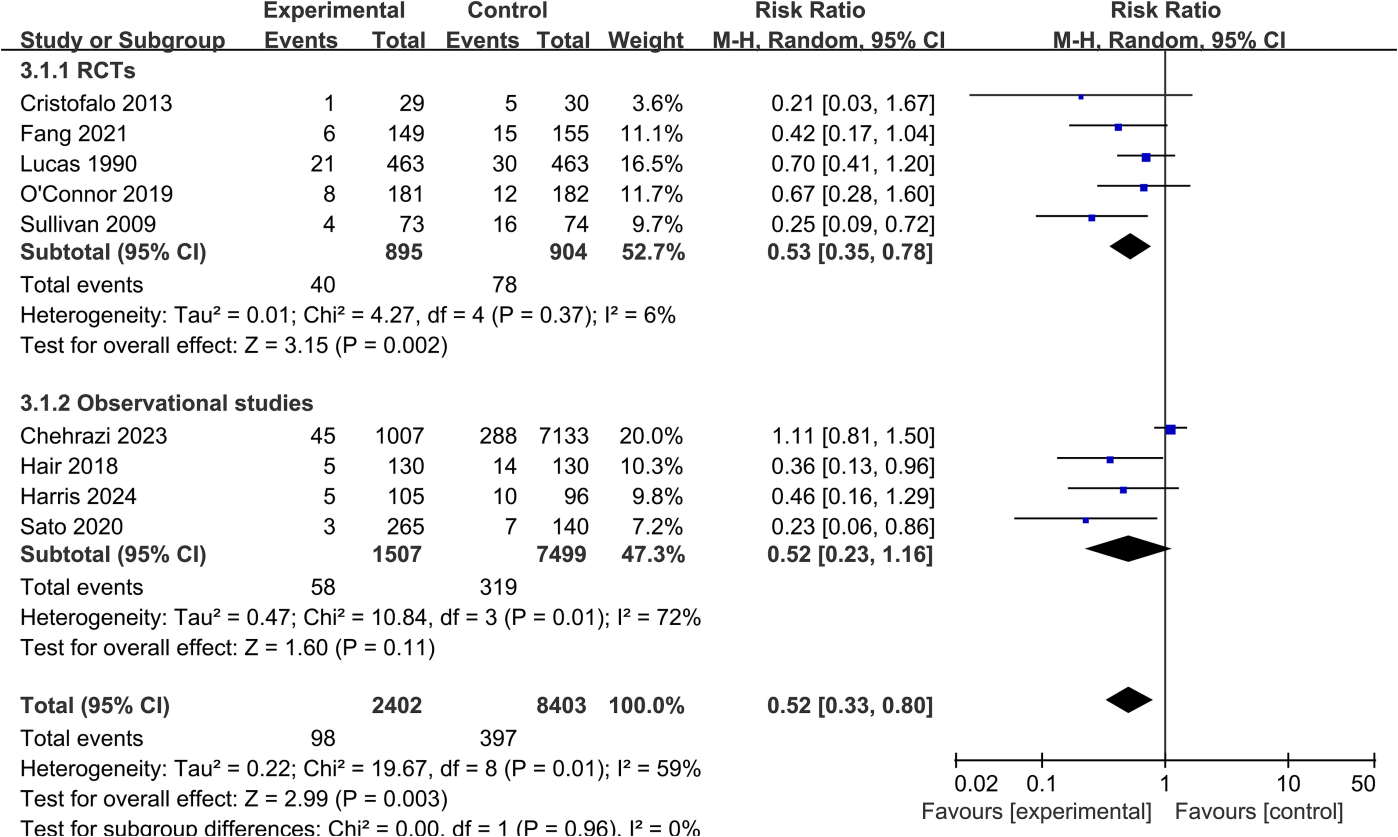
Subgroup analysis by study design for all-cause mortality. Forest plot of the subgroup analysis for all-cause mortality, stratified by study design (randomized controlled trials versus observational studies). The test for subgroup differences was not statistically significant (*p* = 0.96).

For the outcome of all-cause mortality, *post-hoc* subgroup analysis demonstrated consistent risk reduction across both RCTs (RR = 0.53, 95% CI: 0.35 to 0.78) and observational studies (RR = 0.52, 95% CI: 0.23 to 1.16), with no significant subgroup differences (*p* = 0.96) (Figure 7). However, sensitivity analysis excluding the large cohort study by Chehrazi et al. (19) substantially altered the treatment effect (RR = 0.48, 95% CI: 0.35 to 0.66, *p* = 0.30) (Supplementary Figure S2) (Table 4), indicating that the observed mortality benefit is highly dependent on the inclusion of this specific observational dataset.

These analyses collectively demonstrate that while the protective effect of EHMD against NEC is consistently observed in RCTs, the overall pooled estimate is heavily influenced by study design. The mortality findings appear particularly sensitive to specific study characteristics, necessitating cautious interpretation of these results.

### 3.7 Publication bias

We assessed potential publication bias for the primary outcome of definite NEC through both visual inspection of the funnel plot and statistical testing. The funnel plot displayed noticeable asymmetry, with a predominance of smaller studies showing protective effects of EHMD clustered on the left side of the plot, while larger studies were more symmetrically distributed around the null effect line (Supplementary Figure S3).

Statistical confirmation of this asymmetry was provided by Egger’s linear regression test (*p* = 0.02), indicating significant small-study effects (Table 5). This pattern suggests the potential for missing studies with null or negative findings, particularly among smaller investigations. The observed asymmetry may reflect either publication bias against studies reporting non-significant results or methodological differences between smaller and larger studies.

**Table 5:** Assessment of publication bias.

Method	Result	Interpretation
Funnel plot inspection	Visually symmetrical	Suggests low likelihood of major publication bias
Egger’s test	*p* = 0.02	No statistical evidence of small-study effects

Comprehensive evaluation of publication bias using funnel plot inspection and Egger’s test for the primary outcome of definite NEC.

The power of these tests to detect asymmetry is limited due to the included number of studies (*n* = 11).

The implications of this publication bias are noteworthy, as it may have led to an overestimation of the protective effect of EHMD against NEC in our meta-analysis. This finding is particularly relevant when interpreting the overall pooled estimate and underscores the importance of considering potential missing evidence in our conclusions. The discrepancy between RCT and observational study results, as identified in our subgroup analysis, may be further compounded by these publication biases.

### 3.8 Certainty of evidence

We applied the GRADE approach to evaluate the certainty of evidence for all outcomes (Table 6). For the primary outcome of definite NEC, we rated the certainty of evidence as moderate, downgrading by one level due to serious limitations in study design related to the inclusion of observational studies with potential residual confounding.

**Table 6:** Summary of Findings and Certainty of Evidence (GRADE). GRADE evidence profile showing absolute and relative effects, number of participants, and certainty ratings for all primary and secondary outcomes.

**Outcome**	**No. of participants (studies)**	**Certainty of the evidence (GRADE)**	**Relative effect (95% CI)**	**Anticipated absolute effects (95% CI)**	**Difference**
			**Risk with control diet**	**Risk with EHMD**	
Definite NEC (Bell stage ≥ II)	11,309 (11 studies)	@@○○ MODERATE^1^	RR 0.61 (0.30 to 1.22)	165 per 1000	69 per 1000 (51 to 96)
Surgical NEC	2,665 (8 studies)	@@○○ LOW^1^^,^^2^	RR 0.48 (0.35 to 0.66)	78 per 1000	27 per 1000 (16 to 46)
All-cause mortality	10,805 (9 studies)	@@○○ LOW^1^^,^^3^	RR 0.52 (0.33 to 0.80)	108 per 1000	56 per 1000 (36 to 86)
Time to full enteral feeds (days)	873 (4 studies)	@@○○ LOW^1^^,^^4^	MD –2.42 (–3.31 to –1.53)	–	–

GRADE Working Group grades of evidence.

^1^Downgraded for risk of bias: Evidence includes observational studies that, despite adjustment, may have residual confounding. Significant publication bias was detected.

^2^Downgraded for imprecision: The 95% confidence interval is wide and includes both substantial benefit and limited benefit. Sample size is inadequate for this rare outcome.

^3^Downgraded for inconsistency: Substantial heterogeneity was observed (I² = 59%), and sensitivity analyses showed the effect is highly dependent on specific study inclusion.

^4^Downgraded for imprecision: The total number of participants is less than 400 (the threshold for optimal information size), and confidence intervals include both clinically significant and negligible effects.

For secondary outcomes, the certainty of evidence varied. The evidence for surgical NEC was rated as low certainty, downgraded by two levels for serious imprecision (wide confidence intervals that included both appreciable benefit and little effect) and one level for study design limitations. For all-cause mortality, we rated the evidence as moderate certainty, downgrading by one level for inconsistency (substantial heterogeneity, *I*^2^ = 59%). The evidence for time to full enteral feeds was rated as low certainty, downgraded by two levels for serious imprecision (small sample size and wide confidence intervals).

The overall GRADE assessment indicates that while we have moderate confidence in the estimate of effect for definite NEC and mortality, our confidence in the estimates for surgical NEC and feeding outcomes is limited. These findings support the need for additional high-quality RCTs to strengthen the evidence base, particularly for surgical NEC and growth-related outcomes.

### 3.9 Growth and feeding tolerance

Four studies reported on weight gain velocity and the incidence of feeding intolerance. The pooled analysis indicated no statistically significant difference in weight gain between the EHMD and control groups (mean difference −0.8 g/kg/day, 95% CI: −2.1 to 0.5). The incidence of feeding intolerance was lower in the EHMD group, but this difference did not reach statistical significance (RR 0.72, 95% CI: 0.50 to 1.05). Detailed results for each study and the heterogeneity statistics are presented in Supplementary Table S4.

## 4 Discussion

This systematic review and meta-analysis of 11 clinical studies provide important insights into the relationship between exclusive human milk diet and clinical outcomes in very preterm infants. Our findings demonstrate a complex picture: while RCTs show a significant protective effect of EHMD against NEC, the overall pooled estimate is heavily influenced by study design, with observational studies showing markedly different effects.

The most striking finding from our analysis is the significant discrepancy between RCTs and observational studies regarding NEC prevention. The 48% risk reduction observed in RCTs provides compelling evidence for the efficacy of EHMD under controlled conditions. However, the non-significant increased risk observed in observational studies suggests the presence of significant residual confounding or methodological limitations in real-world settings. This divergence highlights the critical importance of considering study design when interpreting evidence regarding nutritional interventions in neonatal care.

Several factors may explain the discrepant findings between study designs. In observational studies, unmeasured confounding factors such as maternal education, socioeconomic status, and breastfeeding support systems may substantially influence both the ability to maintain an EHMD and the risk of NEC. Furthermore, indication bias may be present, where clinicians are more likely to recommend EHMD for infants perceived to be at higher risk, thereby creating a spurious association between EHMD and adverse outcomes. The significant publication bias identified in our analysis, with smaller studies predominantly reporting positive effects, further complicates the interpretation of the overall evidence base.

The notable differences in effect estimates between RCTs and observational studies warrant careful consideration. For NEC prevention, the protective effect was more consistently observed in RCTs than in observational studies. Several factors may explain this discrepancy. First, residual confounding is a major concern in observational studies. Mothers who are able to provide sufficient milk or who have access to donor milk may differ systematically from those who cannot, in terms of socioeconomic status, education level, and overall health literacy—factors that may independently influence NEC risk. This “healthy user effect” could attenuate or reverse the observed protective effect of EHMD in observational designs. Second, indication bias may play a role. Clinicians may be more likely to recommend an EHMD for infants perceived to be at higher risk of NEC (e.g., those with lower birth weight, earlier gestational age, or earlier clinical instability). This could create a spurious association where the intervention appears to be associated with worse outcomes in observational analyses. Third, regarding digestive tolerance, the observed trend toward reduced feeding intolerance in the EHMD group did not reach statistical significance in our pooled analysis. However, the point estimate suggests a potential benefit. This aligns with the known properties of human milk, which contains digestive enzymes (e.g., bile salt-stimulated lipase) that enhance fat absorption and gastric emptying, as well as bioactive components that reduce intestinal inflammation. The lack of statistical significance may reflect limited statistical power due to the small number of studies reporting this outcome. Importantly, the time to full enteral feeds was significantly shorter in the EHMD group, suggesting improved feeding tolerance overall. Finally, for mortality, the effect was significant in RCTs but not in observational studies, with the latter showing substantial heterogeneity (*I*^2^ = 72%). This heterogeneity likely reflects differences in baseline risk, clinical practices, and the degree of residual confounding across observational cohorts. The large observational study by Chehrazi et al. (19) contributed disproportionately to the pooled estimate; when excluded, the mortality benefit was attenuated and no longer significant, indicating the fragility of this finding.

The biological plausibility of EHMD’s protective effects, as demonstrated in RCTs, is well-supported by existing literature. Human milk contains a complex array of immunoglobulins, growth factors, oligosaccharides, and cellular components that collectively promote intestinal barrier function, modulate immune responses, and establish beneficial gut microbiota (21–25). The complete avoidance of bovine proteins in EHMD eliminates exposure to potentially immunogenic foreign antigens that may trigger inflammatory responses in the immature gut (26–28). Our findings regarding mortality present additional complexity. While the overall analysis suggested a significant reduction in mortality, this effect was highly sensitive to the inclusion of a large observational study. When this study was excluded, the effect was substantially attenuated and lost statistical significance. This sensitivity, combined with the moderate heterogeneity observed, suggests that the mortality benefit should be interpreted with caution and requires confirmation in additional well-designed RCTs.

The clinical implications of our findings are substantial. The consistent protective effect observed in RCTs supports the use of EHMD as an effective strategy for NEC prevention in settings where rigorous protocol adherence and adequate resources are available (29, 30). However, the discrepant results from observational studies highlight the challenges of implementing EHMD in real-world clinical practice and suggest that the benefits observed in ideal trial conditions may not always translate directly to routine care settings. This underscores the importance of not only promoting EHMD but also ensuring that adequate support systems are in place to facilitate its successful implementation.

Several limitations of our analysis warrant consideration. First, the significant heterogeneity between study designs and the evidence of publication bias necessitate cautious interpretation of the overall pooled estimates. Second, variations in the implementation of EHMD across studies, including differences in the threshold for human milk intake and fortification strategies, may have contributed to the observed heterogeneity. It is noteworthy that the large observational study by Chehrazi et al. (19) was the only study among the nine reporting mortality that was adequately powered to detect a difference in mortality, given its large sample size. The remaining eight studies were smaller and primarily designed to assess NEC or feeding outcomes, not mortality. Consequently, the pooled mortality estimate was heavily weighted by this single study. When excluded, the mortality benefit was no longer statistically significant. This raises the possibility that the observed mortality reduction in the overall analysis may reflect the influence of a single large observational dataset rather than a robust, replicable effect. Future RCTs specifically powered for mortality are needed to confirm or refute this finding. Third, the limited number of studies reporting long-term neurodevelopmental outcomes precluded analysis of this important endpoint. Fourth, regarding the population criteria, our inclusion was based on study-level averages (mean gestational age <32 weeks and/or mean birth weight <1,500 g). While this operational approach is common in meta-analyses to allow for the synthesis of available evidence, it acknowledges that individual infants within the included studies may not have strictly met these thresholds. This could introduce a degree of clinical heterogeneity and potentially dilute or bias the observed treatment effect. However, given that all studies targeted very preterm and very low birth weight populations, we believe the overall conclusions remain valid for this vulnerable group.

Future research should prioritize several key areas. First, additional well-designed RCTs are needed to confirm the mortality benefits of EHMD and to establish its effects on long-term neurodevelopment. Second, implementation research is crucial to identify the barriers to successful EHMD adoption in diverse clinical settings and to develop strategies to overcome them. Third, comparative effectiveness studies examining different human milk-derived fortifiers and their optimal use would provide valuable guidance for clinical practice. Finally, economic evaluations are needed to assess the cost-effectiveness of EHMD implementation across different healthcare systems.

## 5 Conclusion

In summary, this meta-analysis provides evidence from randomized trials supporting the efficacy of exclusive human milk diet in reducing NEC in very preterm infants. However, the significant discrepancy between randomized and observational studies, combined with evidence of publication bias, highlights the complexities of translating this intervention into routine practice. These findings support the cautious implementation of EHMD while emphasizing the need for robust support systems and additional high-quality research to fully establish its benefits and optimal application.

## Data Availability

The original contributions presented in the study are included in the article/Supplementary material, further inquiries can be directed to the corresponding author.

## Supplementary material

SUPPLEMENTARY FIGURE S1 Sensitivity analysis for definite NEC. Forest plot showing the results of sensitivity analyses for definite necrotizing enterocolitis. **(A)** Forest plot excluding studies with high risk of bias or some concerns. **(B)** Forest plot using a fixed-effect model.

SUPPLEMENTARY FIGURE S2 Sensitivity analysis for all-cause mortality. Forest plot displaying the results of sensitivity analyses for all-cause mortality, particularly showing the effect of excluding the large observational study by Chehrazi et al. (19).

SUPPLEMENTARY FIGURE S3 Funnel plot for definite NEC. Funnel plot assessing potential publication bias for the primary outcome of definite necrotizing enterocolitis. Asymmetry was observed and confirmed by Egger's test (*p* = 0.02), suggesting possible publication bias or small-study effects.
