# Supplementary figures and images for: Efficacy and heterogeneity: an exclusive human milk diet for necrotizing enterocolitis prevention in very preterm infants—a systematic review and meta-analysis of 11 studies
Source: Front Nutr. 2026 May 20;13:1768141. doi: 10.3389/fnut.2026.1768141 (PMC13229633; doi:10.3389/fnut.2026.1768141)

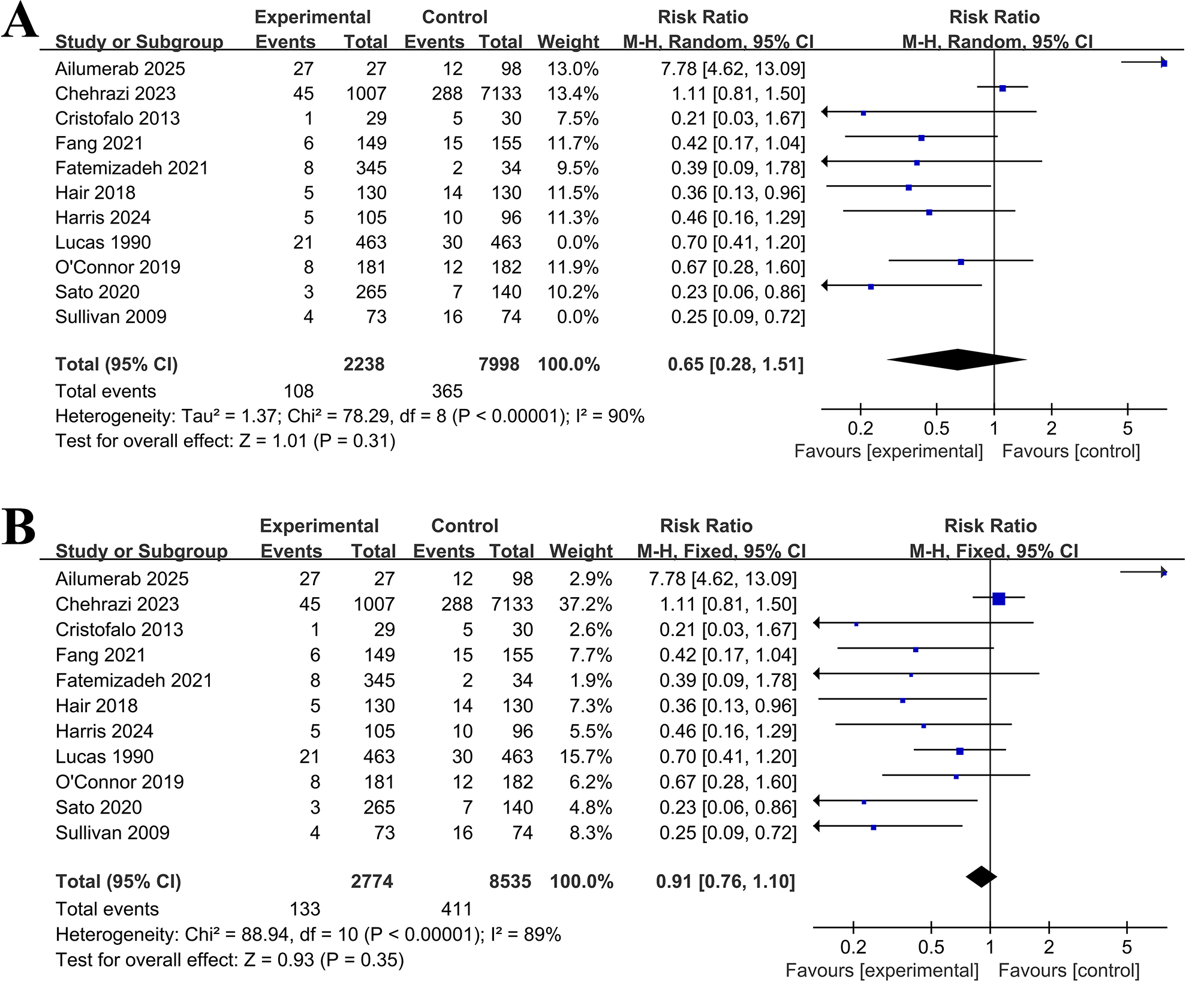

Supplement: SUPPLEMENTARY FIGURE S1 — Sensitivity analysis for definite NEC. Forest plot showing the results of sensitivity analyses for definite necrotizing enterocolitis. (A) Forest plot excluding studies with high risk of bias or some concerns. (B) Forest plot using a fixed-effect model. [file Image_1.JPEG]

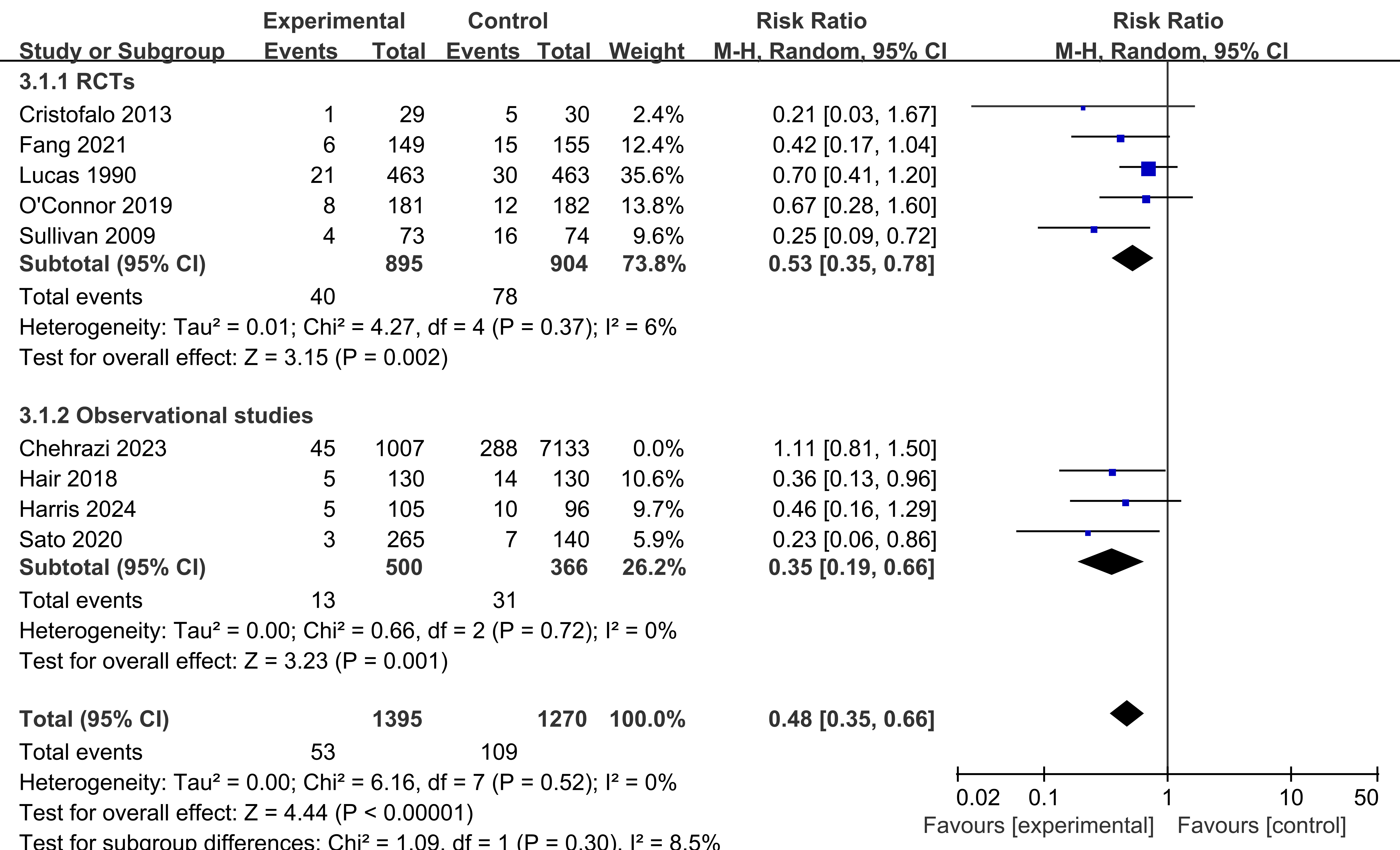

Supplement: SUPPLEMENTARY FIGURE S2 — Sensitivity analysis for all-cause mortality. Forest plot displaying the results of sensitivity analyses for all-cause mortality, particularly showing the effect of excluding the large observational study by Chehrazi et al. (19). [file Image_2.JPEG]

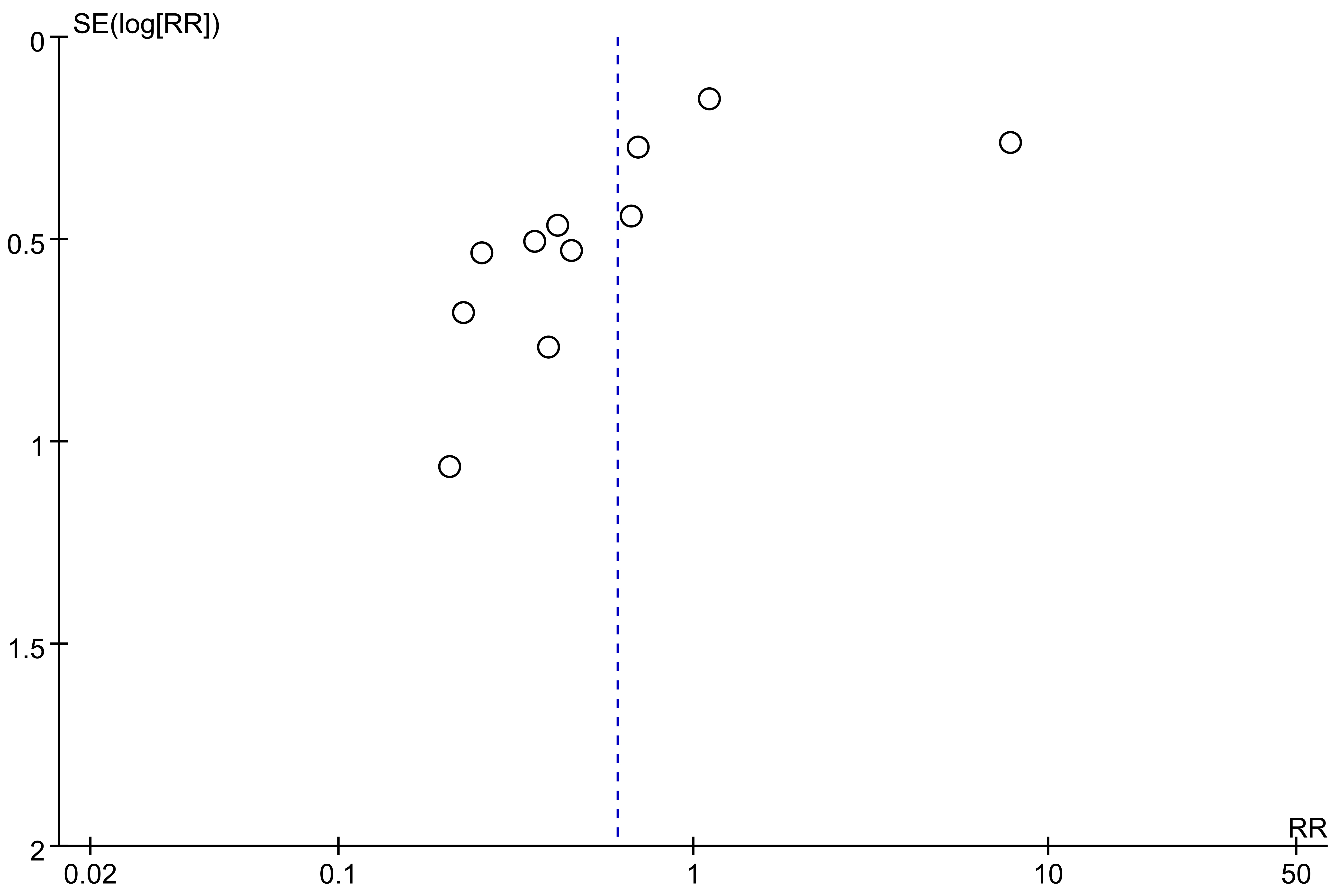

Supplement: SUPPLEMENTARY FIGURE S3 — Funnel plot for definite NEC. Funnel plot assessing potential publication bias for the primary outcome of definite necrotizing enterocolitis. Asymmetry was observed and confirmed by Egger's test (p = 0.02), suggesting possible publication bias or small-study effects. [file Image_3.JPEG]
