# Supplementary material for: Efficacy and heterogeneity: an exclusive human milk diet for necrotizing enterocolitis prevention in very preterm infants—a systematic review and meta-analysis of 11 studies
Source: Front Nutr. 2026 May 20;13:1768141. doi: 10.3389/fnut.2026.1768141 (PMC13229633; doi:10.3389/fnut.2026.1768141)
Supplement: Supplementary file 4 [file Table_1.docx]

**Supplementary Table S1. Detailed Search Strategies by Database**

This supplementary table provides the complete search strategies used for each electronic database (PubMed, Embase, Cochrane CENTRAL, and CINAHL), including the specific search terms, syntax, and number of records retrieved from each database. Additional search methods such as clinical trial registry searches and manual reference checking are also described.

| **Database** | **Search Strategy** | **Results** | **Date Searched** |
| --- | --- | --- | --- |
| **PubMed** | 1. "Necrotizing Enterocolitis"[Mesh] OR "Enterocolitis, Necrotizing"[tw] OR "Necrotising Enterocolitis"[tw] OR NEC[tw]  2. "Milk, Human"[Mesh] OR "Breast Feeding"[Mesh] OR "human milk"[tw] OR "breast milk"[tw] OR "mother's milk"[tw] OR "donor milk"[tw]  3. "Infant Formula"[Mesh] OR "formula"[tw] OR "bovine milk"[tw] OR "cow's milk"[tw] OR "preterm formula"[tw]  4. "Infant, Premature"[Mesh] OR "Infant, Very Low Birth Weight"[Mesh] OR "preterm infant"[tw] OR "very low birth weight"[tw] OR VLBW[tw]  5. #1 AND #2 AND #3 AND #4  6. #5 NOT (("Animals"[Mesh] NOT "Humans"[Mesh])) | 1,842 | October 15, 2025 |
| **Embase** | 1. 'necrotizing enterocolitis'/exp OR 'necrotizing enterocolitis':ti,ab OR 'necrotising enterocolitis':ti,ab OR 'nec':ti,ab  2. 'breast milk'/exp OR 'breast feeding'/exp OR 'human milk':ti,ab OR 'breast milk':ti,ab OR 'donor milk':ti,ab OR 'banked milk':ti,ab  3. 'infant formula'/exp OR 'formula':ti,ab OR 'bovine milk':ti,ab OR "cow's milk":ti,ab OR 'preterm formula':ti,ab  4. 'premature infant'/exp OR 'very low birth weight infant'/exp OR 'preterm infant':ti,ab OR 'premature infant':ti,ab OR 'vlbw':ti,ab OR 'very low birth weight':ti,ab  5. #1 AND #2 AND #3 AND #4  6. #5 NOT ([animals]/lim NOT [humans]/lim) | 2,156 | March 15, 2024 |
| **Cochrane CENTRAL** | 1. (necrotizing enterocolitis OR necrotising enterocolitis OR NEC):ti,ab,kw  2. (human milk OR breast milk OR donor milk OR banked milk):ti,ab,kw  3. (formula OR bovine milk OR cow's milk OR preterm formula):ti,ab,kw  4. (preterm infant OR premature infant OR VLBW OR very low birth weight):ti,ab,kw  5. #1 AND #2 AND #3 AND #4 | 892 | March 16, 2024 |
| **CINAHL** | 1. (MH "Necrotizing Enterocolitis") OR TI ("Necrotizing Enterocolitis" OR "Necrotising Enterocolitis" OR NEC) OR AB ("Necrotizing Enterocolitis" OR "Necrotising Enterocolitis" OR NEC)  2. (MH "Breast Feeding") OR (MH "Milk, Human") OR TI ("human milk" OR "breast milk" OR "donor milk") OR AB ("human milk" OR "breast milk" OR "donor milk")  3. (MH "Infant Formula") OR TI (formula OR "bovine milk" OR "cow's milk") OR AB (formula OR "bovine milk" OR "cow's milk")  4. (MH "Infant, Premature") OR (MH "Infant, Very Low Birth Weight") OR TI ("preterm infant" OR "premature infant" OR VLBW) OR AB ("preterm infant" OR "premature infant" OR VLBW)  5. #1 AND #2 AND #3 AND #4 | 1,127 | March 16, 2024 |

All searches were performed from database inception to March 2024.No language restrictions were applied. Titles/abstracts of non-English records were translated using automated tools for initial screening. Full texts of potentially eligible studies were translated professionally. Search strategies were peer-reviewed by a medical librarian using the PRESS checklist.

Field codes: [Mesh] = Medical Subject Headings, [tw] = text words, ti = title, ab = abstract, kw = keywords, /exp = exploded term

Boolean operators: AND = intersection, OR = union, NOT = exclusion

All database results were exported to EndNote X20 for deduplication and management

Additional Search Methods:

Clinical Trial Registries: ClinicalTrials.gov and WHO ICTRP were searched using terms "necrotizing enterocolitis" AND ("human milk" OR "donor milk")

Manual Searching: Reference lists of all included studies and relevant systematic reviews were examined

Grey Literature: Conference proceedings from Pediatric Academic Societies (2020-2024) were reviewed
