# Supplementary material for: Efficacy and heterogeneity: an exclusive human milk diet for necrotizing enterocolitis prevention in very preterm infants—a systematic review and meta-analysis of 11 studies
Source: Front Nutr. 2026 May 20;13:1768141. doi: 10.3389/fnut.2026.1768141 (PMC13229633; doi:10.3389/fnut.2026.1768141)
Supplement: Supplementary file 5 [file Table_2.docx]

**Supplementary Table S2. Risk of Bias and Quality Assessment of Included Studies**

This supplementary table presents the detailed results of methodological quality assessment for all included studies. Part A shows the risk of bias assessment for randomized controlled trials using the Cochrane RoB 2.0 tool across five domains, while Part B displays the quality assessment for observational studies using the Newcastle-Ottawa Scale across three domains with total scores and quality ratings.

| **Part A: Randomized Controlled Trials (Assessed using the Cochrane RoB 2.0 tool)** | | | | | | |
| --- | --- | --- | --- | --- | --- | --- |
| **Study (Year)** | **Randomization Process** | **Deviations from Intended Interventions** | **Missing Outcome Data** | **Measurement of the Outcome** | **Selection of Reported Result** | **Overall Bias** |
| Cristofalo (2013) | Low | Low | Low | Low | Low | **Low** |
| O'Connor (2019) | Low | Low | Low | Low | Low | **Low** |
| Sullivan (2009) | Low | Some Concerns | Low | Low | Low | **Some Concerns** |
| Lucas (1990) | Some Concerns | Some Concerns | Low | Low | Low | **Some Concerns** |
| Fang (2021) | Low | Low | Low | Low | Low | **Low** |

| **Part B: Observational Studies (Assessed using the Newcastle-Ottawa Scale - NOS)** | | | | | | |
| --- | --- | --- | --- | --- | --- | --- |
| **Study (Year)** | **Design** | **Selection (Max 4)** | **Comparability (Max 2)** | **Outcome/Exposure (Max 3)** | **Total Score (Max 9)** | **Quality Rating** |
| Hair (2018) | Cohort | 4 | 2 | 3 | **9** | High |
| Sato (2020) | Cohort | 3 | 2 | 3 | **8** | High |
| Fatemizadeh (2021) | Cohort | 3 | 1 | 3 | **7** | High |
| Chehrazi (2023) | Cohort | 3 | 1 | 2 | **6** | Moderate |
| Harris (2024) | Cohort | 4 | 2 | 2 | **8** | High |
| Ailumerab (2025) | Case-Control | 4 | 2 | 2 | **8** | High |

Assessment Criteria and Interpretation:

Cochrane RoB 2.0:

Low risk: Plausible bias unlikely to seriously alter the results

Some concerns: Plausible bias that raises some doubt about the results

High risk: Plausible bias that seriously weakens confidence in the results

Newcastle-Ottawa Scale:

High quality: 7-9 stars

Moderate quality: 4-6 stars

Low quality: 0-3 stars

Key Findings:

Among RCTs, 3 studies (60%) were judged as low risk of bias overall

2 RCTs (40%) had some concerns, primarily related to deviations from intended interventions

Among observational studies, 5 (83%) were rated as high quality (NOS score ≥7)

1 cohort study (17%) was rated as moderate quality (NOS score=6), mainly due to inadequate control for confounding factors

The overall methodological quality of the included studies was satisfactory, with the majority demonstrating low risk of bias or high quality ratings.
