# Supplementary material for: Efficacy and heterogeneity: an exclusive human milk diet for necrotizing enterocolitis prevention in very preterm infants—a systematic review and meta-analysis of 11 studies
Source: Front Nutr. 2026 May 20;13:1768141. doi: 10.3389/fnut.2026.1768141 (PMC13229633; doi:10.3389/fnut.2026.1768141)
Supplement: Supplementary file 6 [file Table_3.docx]

**Supplementary Table S3. Detailed Sensitivity Analysis: Pooled Effect Estimates for Primary and Secondary Outcomes Under Different Scenarios**

Comprehensive sensitivity analysis results showing pooled effect estimates under different scenarios including exclusion of high risk studies and alternative statistical models.

| **Outcome** | **Analysis Scenario** | **Studies Removed / Analysis Method** | **Number of Studies (Participants)** | **Pooled RR/MD (95% CI)** | **I²** | **Conclusion** |
| --- | --- | --- | --- | --- | --- | --- |
| **Definite NEC** | **Primary Analysis** | **None** | **11 (11,030)** | **RR 0.91 (0.76-1.10)** | **45%** | **Reference** |
|  | Sensitivity 1 | High/unclear risk of bias | 8 (1,963) | RR 0.65 (0.28-1.51) | 42% | Direction consistent, wider CI |
|  | Sensitivity 2 | Fixed-effect model | 11 (11,030) | RR 0.91 (0.76-1.10) | 45% | Robust |
|  | Sensitivity 3 | Largest study by weight | 10 (2,890) | RR 0.48 (0.34-0.67) | 38% | Effect strengthened |
| **All-cause Mortality** | **Primary Analysis** | **None** | **9 (10,805)** | **RR 0.52 (0.33-0.80)** | **59%** | **Reference** |
|  | Sensitivity 1 | High/unclear risk of bias | 6 (1,019) | RR 0.67 (0.44-1.02) | 0% | Effect attenuated |
|  | Sensitivity 2 | Fixed-effect model | 9 (10,805) | RR 0.58 (0.45-0.75) | 59% | Robust |
|  | Sensitivity 3 | Excluding Chehrazi 2023 | 8 (1,019) | RR 0.48 (0.35-0.66) | 0% | Effect maintained |

The primary analysis for all outcomes used a random-effects model (DerSimonian and Laird). High/unclear risk of bias studies were those rated as "some concerns" or "high risk" in the RoB 2.0 tool for RCTs, or a NOS score ≤ 6 for observational studies. Robust indicates that the conclusion (statistical significance and direction of effect) did not change compared to the primary analysis. The largest study by weight in the primary outcome analysis was Chehrazi et al. (2023).

CI, confidence interval; MD, mean difference; NEC, necrotizing enterocolitis; RR, risk ratio.
