# Supplementary material for: Efficacy and heterogeneity: an exclusive human milk diet for necrotizing enterocolitis prevention in very preterm infants—a systematic review and meta-analysis of 11 studies
Source: Front Nutr. 2026 May 20;13:1768141. doi: 10.3389/fnut.2026.1768141 (PMC13229633; doi:10.3389/fnut.2026.1768141)
Supplement: Supplementary file 7 [file Table_4.docx]

**Supplementary Table S4. Meta-analysis of Growth and Feeding Tolerance Outcomes in Very Preterm Infants**

| **Study (Year)** | **Weight Gain Velocity (g/kg/day)** | | | **Feeding Intolerance Incidence** | | |
| --- | --- | --- | --- | --- | --- | --- |
|  | **EHMD Group (Mean ± SD)** | **Control Group (Mean ± SD)** | **Weight (%)** | **EHMD Events/Total** | **Control Events/Total** | **Weight (%)** |
| **Cristofalo (2013)** | 15.1 ± 3.0 | 15.9 ± 3.4 | 24.9% | 7 / 29 | 11 / 30 | 26.3% |
| **O'Connor (2019)** | 14.9 ± 2.9 | 15.6 ± 3.1 | 26.7% | 10 / 181 | 15 / 182 | 31.8% |
| **Sullivan (2010)** | 15.8 ± 3.3 | 16.5 ± 3.7 | 23.5% | 5 / 73 | 9 / 74 | 24.5% |
| **Fang (2021)** | 15.4 ± 3.2 | 16.1 ± 3.6 | 24.9% | 9 / 149 | 14 / 155 | 17.4% |
| **Pooled Effect (95% CI)** | **MD = -0.8 (-2.1 to 0.5)** | | **100%** | **RR = 0.72 (0.50 to 1.05)** | | **100%** |
| **Heterogeneity** | **I² = 32%, p = 0.21** | | | **I² = 18%, p = 0.30** | | |

CI, confidence interval; EHMD, exclusive human milk diet; MD, mean difference; RR, risk ratio; SD, standard deviation.

Notes:

Weight Gain Velocity was analyzed as a continuous outcome. The pooled Mean Difference (MD) and 95% CI were calculated using the inverse variance method under a random-effects model.

Feeding Intolerance was analyzed as a dichotomous outcome, defined as gastric residuals >50% of feed volume and/or abdominal distension requiring feed withholding. The pooled Risk Ratio (RR) and 95% CI were calculated using the Mantel-Haenszel method under a random-effects model.

Weight (%) represents the contribution of each study to the pooled estimate in the meta-analysis.

Heterogeneity was assessed using the I² statistic and Cochran's Q test (p-value).

The four studies included in this analysis were those that reported data on both growth and feeding tolerance outcomes among the 11 included studies.
